# Draft genome sequences of three *Serratia liquefaciens* strains isolated from vegetables in South Africa

**DOI:** 10.1128/mra.00772-24

**Published:** 2025-02-11

**Authors:** Mariska S. Kleyn, Muiz O. Akinyemi, Carlos Bezuidenhout, Rasheed A. Adeleke

**Affiliations:** 1 Unit for Environmental Sciences and Management, North-West University, Potchefstroom, South Africa; 2 Leeds Institute of Health Sciences, University of Leeds, Leeds, United Kingdom; Indiana University, Bloomington, Bloomington, Indiana, USA

**Keywords:** endophytes, antibiotic resistance, *Serratia*, vegetable, food safety, genomes

## Abstract

Strains of the Gram-negative, facultatively anaerobic, rod-shaped *Serratia* genus are plant endophytes. Here, we described three draft genome sequences of *Serratia liquefaciens* isolated in South Africa from the leaves of tomatoes and lettuce.

## ANNOUNCEMENT

*Serratia* is found in various niches of plants including the leaves of vegetables (1). Bioprospecting vegetable ecosystem may yield interesting endophytes, with high potential for plant growth promotion. In this context, we present the draft genome of three *Serratia liquefaciens* strains, considered as potential plant endophytes, isolated from tomato and lettuce leaf at harvest.

About 2 g of leaf tissues of healthy single tomatoes and lettuce plant were surface sterilized using 85% ethanol and 2.5% sodium hypochlorite. Afterward, they were crushed, suspended (1 g) in 20 mL of M17 broth, and then incubated at 37°C for 24 hours. Thereafter, 500 µL of the suspension was cultured and restreaked (×2) until pure cultures were obtained on M17 agar incubated at 37°C for 24 hours.

Genomic DNA was extracted from single-colony cultures grown on M17 agar for 24 hours at 37°C using the Quick-DNA Fungal/bacterial Miniprep Kit (Zymo Research). Subsequently, genomic library was prepared using the Illumina TruSeq Nano DNA library preparation kit provided by Illumina. Paired-end sequencing (2 × 150 bp) was performed using Illumina NovaSeq 6000. Quality assessment of sequence reads was carried out using FastQC v.0.12.0 (1). Adapter trimming, quality filtering, and pruning were performed using fastp v.0.23.4 (2). Filtered paired-end reads were *de novo* assembled using SPAdes v.3.15.3 (3). Genome completeness was evaluated using CheckM v.1.0.18 (4). Identification of closely related reference genomes and calculation of orthologous average nucleotide identity were performed using Mash software v.2.3 (5). Genome annotation was performed using Prokka v.1.14.6 (6), with minimum contig length set to 200. ABRicate v.1.0.1 (7) was used to examine virulence determinants and pathogenic genes by aligning reads to the Virulence Factors Database (8) and PHI-base v.4.14 (9), respectively. Simultaneously, resistance-related genes were predicted by aligning reads against the Comprehensive Antibiotic Resistance Database v.4.0.0 (10), ResFinder v.4.0 (11), and AMRFinderPlus v.3.10.42 (12) database. Insertion sequence (IS) was identified using ISEScan v.1.7.2.3 (13). Single-copy IS elements without perfect terminal inverted repeats or shorter than 400 bp were excluded. The resulting outputs are presented in Table 1, while annotations, virulence, and resistance data are available on Figshare. Default parameters was used for all software unless otherwise specified.

**TABLE 1:** Details on the whole genomes of *Serratia liquefaciens*

	Isolates		
	M17KBT1B	M17VKL4D	M17VKL4B
Source	Tomato leaf	Lettuce leaf	Lettuce leaf
Sampling locality	North West province, South Africa	Gauteng province, South Africa	Gauteng province, South Africa
Global positioning system	32°45′31.867″ N, 97°19′43.702 E	26°16′14.733″ S, 28°6′44.164 E	26°16′14.733″ S, 28°6′44.164 E
Read count (paired)	5,438,508	6,189,792	6,780,931
Coverage	315	360.2	394.8
Completeness	100	100	100
Contamination	0.49	0.45	0.45
Number of scaffolds	21	20	21
Smallest scaffold	501	533	533
Largest scaffold	2,008,815	1,702,344	1,004,825
Average scaffold length	129,188	257,171	244,915
N50	699,025	1,004,825	698,631
L50	2	2	3
Number of contigs	24	25	27
Size (bp)	5,167,520	5,143,431	5,143,222
Guanine cytosine content (%)	55.4	55.4	55.4
Protein-coding features	4,870	4,862	4864
Non-coding features	99	105	110
Average nucleotide identity	98.83	98.83	98.8
Mash distance	0.00944662	0.00936594	0.00936594
Antibiotic resistance genes			
CARD	CRP, msbA, rsmA, Kpne_KpnH, emrR	CRP, msbA, rsmA, Kpne_KpnH, emrR	CRP, msbA, rsmA, Kpne_KpnH, emrR
ResFinder	OqxB	OqxB	OqxB
AMRFinderPlus	ampC, qnrE, blaSPR	ampC, qnrE, blaSPR	ampC, qnrE, blaSPR
Virulence factors	11	11	11
Pathogenicity related	179	179	180
Insertion sequences	2	3	3
BioSample accession number	SAMN38749209	SAMN38749214	SAMN38749217
Sequence Read Archive	SRR28883055	SRR28883054	SRR28883053

The assembled genomes were characterized by CheckM as 100% complete with 0.45%–0.49% contamination. The genome sizes for M17KBT1B, M17VKL4B, and M17VKL4D were 5,167,520, 5,143,222, and 5,143,431 bp with 24, 25, and 27 contigs, respectively. All strains had a GC content of 55.4%.The taxonomic classification of M17KBT1B, M17VKL4D, and M17VKL4B closely matches *S. liquefaciens* (GCA_000422085.1), with an average nucleotide identity value of approximately 98.8%. These strains contain 4,870, 4,862, and 4,864 protein-coding features, respectively. Each strain was predicted to harbor nine resistance-related genes, including EmrR (linked to fluoroquinolone resistance) and OqxB (associated with disinfectant resistance). All strains were predicted to possess 11 native *Serratia* virulence-related genes, with motility genes being the most abundant (*n* = 5). Additionally, at least 179 pathogenicity-related genes were predicted in all strains, except for the EepR gene, which was present only in M17VKL4B. The IS family IS630 and IS256 were unique to M17VKL4D and M17VKL4B, respectively.

## Data Availability

This Whole Genome Shotgun project has been deposited at DDBJ/ENA/GenBank under accession number PRJNA1050647. The 3 genomes reported in our study are part of a set of 10 genomes that were sequenced. The BioSample details for the respective strains are provided in Table 1. The genome annotation in GenBank format predicted resistance, and virulence genes in the draft genome sequences have been deposited at Figshare DOI: https://doi.org/10.6084/m9.figshare.25713843
